# Sudden Consecutive Bilateral Amaurosis Secondary to Central Retinal Artery Occlusion After Kidney Transplantation

**DOI:** 10.7759/cureus.34204

**Published:** 2023-01-25

**Authors:** Daniela Rego-Lorca, Bárbara Burgos-Blasco, Francisco Javier Moreno-Morillo, Alicia Valverde-Megías, José Ignacio Fernández-Vigo

**Affiliations:** 1 Ophthalmology, Sant Pau Hospital, Barcelona, ESP; 2 Ophthalmology, Clinico San Carlos Hospital, Madrid, ESP; 3 Ophthalmology, Infanta Sofía University Hospital, Madrid, ESP

**Keywords:** acute blindness, transplantation, postoperative complications, stroke, central retinal artery occlusion

## Abstract

Central retinal artery occlusion (CRAO) is a medical emergency, considered a stroke equivalent by the American Heart Association. There are a few reported cases of bilateral CRAO, most of them occurring in the context of a systemic predisposing condition. We present a case of bilateral CRAO following kidney transplantation. This 58-year-old man suffered CRAO in the right eye 24 hours after having kidney transplantation surgery. Treatment with an intravenous bolus of high-dose corticosteroids and full-dose anticoagulation therapy was initiated. However, 48 hours later, the patient suffered contralateral CRAO, resulting in irreversible bilateral amaurosis. CRAO is a rare but devastating complication of non-ophthalmological surgery and must be considered in postoperative patients with visual complaints. CRAO may have different causal mechanisms, but due to the similarity of their clinical manifestations, accurate etiology is not always easy to establish. Given the importance of an early diagnosis, all physicians should know about its risk factors and be aware of how patients with suspected CRAO must be rapidly referred for general and ophthalmological evaluation.

## Introduction

Central retinal artery occlusion (CRAO) is a medical emergency, not only due to severe and permanent associated visual loss but also because of the acute systemic implications. CRAO is considered a stroke equivalent by the American Heart Association (AHA) and the American Stroke Association (ASA), patients who suffer a CRAO are at increased risk of cerebral or myocardial infarction within the following weeks [1]. Thus, they should be rapidly referred for general evaluation and stroke risk assessment. On top of that, ophthalmological consequences are devastating, with most of these patients having a visual acuity of 20/400 or worse [2].

CRAO has a global incidence of around 1 in 100,000 people. According to its etiology, it can be classified as arteritic CRAO and non-arteritic CRAO, the latter representing more than 90% of all cases [1]. Non-arteritic CRAO is associated with cardiovascular risk factors and artery disease, and it is typically caused by embolism. Up to 75% of these emboli are made of cholesterol (Hollenhorst plaque), and the ipsilateral internal carotid artery is their most common source, followed by the aortic arch and the heart [2]. Approximately 5% of CRAOs are found to be arteritic. These generally occur in the context of giant cell arteritis (GCA), but, rarely, they can also appear associated with other inflammatory vascular diseases [2].

Sudden painless visual loss is the usual form of presentation of CRAO. In arteritic CRAO in the context of GCA, ophthalmic manifestations are normally accompanied by other symptoms such as anorexia, weight loss, jaw claudication, scalp tenderness, headache, or myalgias, but up to 21% of patients with GCA may not have any systemic symptoms [3]. Regardless of etiology, typical fundoscopic findings in CRAO include macular cherry-red spots, retinal opacity, arterial attenuation, and optic disc edema. Optical coherence tomography (OCT) generally reveals the increased thickness of the inner retina and optic disc swelling in the acute phase, followed, after some weeks, by retinal atrophy and thinning [2]. Given the similarity of their clinical manifestations, accurate etiology of CRAO might not always be easy to establish.

We present the case of a patient who suffered CRAO in his left eye the first day after kidney transplantation surgery and subsequent CRAO in the right eye 48 hours later, resulting in bilateral amaurosis.

## Case presentation

A 58-year-old man with stage 5 chronic kidney disease on hemodialysis secondary to IgA nephropathy was admitted to the hospital to undergo kidney transplantation. He had a history of high blood pressure with stage 1 hypertensive retinopathy and atrial fibrillation requiring anticoagulation therapy with acenocoumarol, which had been switched to prophylactic dose sodium heparin one week before surgery. No incidents were reported throughout the intervention, the patient was stable and no hypotensive attacks occurred.

**Figure 1:**
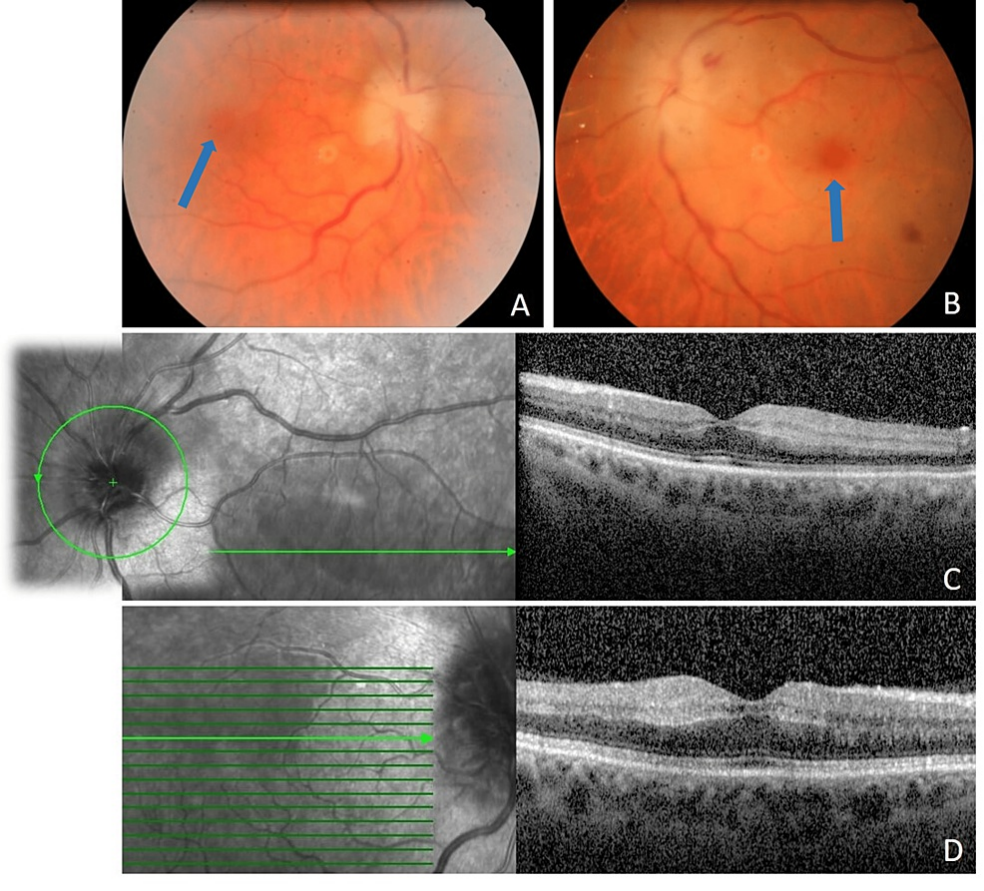
(A and B) Bilateral retinography three days after kidney transplantation surgery showing typical findings of central retinal artery occlusion: optic nerve edema with indistinct margins, macular cherry-red spots (arrows), retinal opacity, arterial attenuation, and intraretinal hemorrhages. (C and D) Macular OCT showing whitening and increased thickness of the inner retinal layers and optic disc swelling in both eyes. OCT, optical coherence tomography

The day after transplantation surgery, the patient complained of sudden, painless visual loss in his left eye (Oculus Sinister or OS). On examination, best-corrected visual acuity (BCVA) was 20/20 in the right eye (Oculus Dexter or OD) and amaurosis in OS. Anterior segment examination showed a clear cornea with a minimum grade of nuclear sclerosis in both eyes. Intraocular pressure (IOP) was within normal limits in both eyes. OS pupil presented a relative afferent pupillary defect. Posterior segment examination of OS revealed optic-nerve-head (ONH) pallor and edema with indistinct margins, macular cherry-red spot, retinal opacity, arterial attenuation, and intraretinal hemorrhages along the inferior vascular arcade and throughout the periphery of retinal parenchyma (Figure 1B). 

Examination of OD was unremarkable. OS macular OCT showed increased thickness of the inner retina and optic disc swelling (Figure 1C). Fluorescein angiography was discouraged due to advanced chronic kidney disease.

Blood analysis showed erythrocyte sedimentation rate (ESR) 64 mm/hour (normal range 0-22 mm/hour) and C-reactive protein (CRP) levels 14.5 mg/L (normal values <10 mg/L). Complete neurological evaluation and head computed tomography (CT) scan did not show any abnormalities. Echo-Doppler of the orbit showed normal blood flow in the ophthalmic artery and that of the supra-aortic vessels and temporal arteries revealed no significant stenosis and no signs of arteritis. A transthoracic echocardiogram demonstrated normal heart anatomy and systolic function within normal limits.

Although increased acute phase reactants levels could be related to prior kidney transplantation surgery, as arteritic CRAO could not be ruled out, treatment with an intravenous bolus of high-dose corticosteroid (methylprednisolone 1 g) was initiated, and anticoagulation therapy with acenocoumarol was restarted. ESR and CRP levels rapidly decreased after corticosteroid therapy onset.

However, 48 hours later, the patient complained of visual loss in the contralateral eye. Visual acuity was now light perception (OD) and amaurosis (OS), progressing in the following hours to bilateral amaurosis. Examination of OS remained as mentioned before, and a new evaluation of OD revealed ONH edema with indistinct margins, macular cherry-red spots, and retinal opacity (Figure 1A). OD macular OCT, similar to previous OS OCT, showed increased thickness of the inner retina and optic nerve swelling (Figure 1D).

Based on bilateral CRAO with associated anterior ischemic optic neuropathy, and the striking visual loss resulting in bilateral amaurosis, ophthalmic artery occlusion was suspected. However, as mentioned earlier, echo-Doppler of the orbit revealed normal blood flow in both ophthalmic arteries, so the final diagnosis was bilateral CRAO.

**Figure 2:**
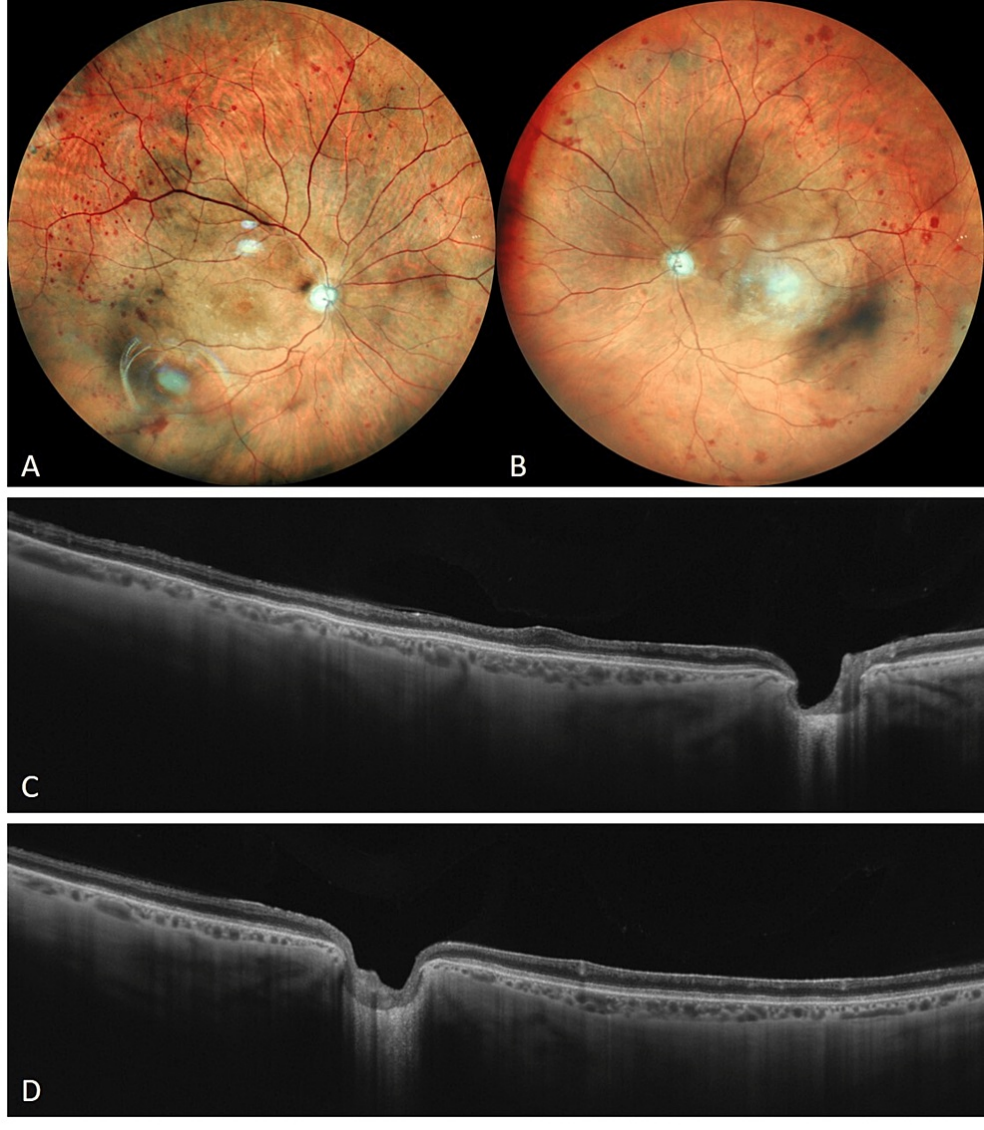
(A and B) Bilateral retinography at a two-year follow-up revealing arteriolar attenuation (more evident in OS), atrophic parenchyma with scattered hemorrhages, and excavated optic disc in both eyes. (C and D) Macular OCT showing a thinner atrophic retina and almost absence of the internal layers in both eyes.

At a two-year follow-up, bilateral retinography showed hemorrhages throughout the atrophic retinal parenchyma and excavated optic disc in both eyes (Figures 2A-2B).

Bilateral OCT demonstrated retinal atrophy and thinning, with almost absent inner retinal layers (Figures 2C-2D). Visual acuity remained bilateral amaurosis, and no signs of complications, such as abnormal neovascularization or neovascular glaucoma, were found. The absence of visual fixation hindered obtaining better-quality images.

## Discussion

Devastating visual loss due to CRAO is well-known by ophthalmologists. The most important factor concerning the visual outcome in CRAO is time. Experimental research in primates has demonstrated no detectable damage within 97 minutes of the occlusion, and different recovery rates when ischemia lasts between two and four hours [4]. More than four hours of CRAO seem to inevitably result in irreversible extensive retinal damage. This experiment also showed that the more time the occlusion lasts, the longer it takes for visual improvement to begin.

Multiple therapies have been tried to attempt to restore retinal circulation. Some of them aimed at decreasing IOP, thus increasing retinal arteries perfusion pressure, such as intravenous acetazolamide and mannitol, topical IOP-lowering medications, or anterior chamber paracentesis [2]. Ocular massage is performed trying both, to decrease IOP and dislodge a potential thrombus or embolus. Regarding dislodging of the embolus, laser embolectomy with neodymium-doped yttrium aluminum garnet (Nd:YAG) laser has also been attempted in a few cases, although around 50% of these patients were affected by vitreous hemorrhage as a complication [1]. Thrombolysis using intravenous or intra-arterial tissue plasminogen activator has also been tried, but due to opposed reported outcomes and high incidence of adverse events, it remains controversial [1]. Moreover, some authors defend surgical removal of retinal artery embolus when visible, and others have proposed pars plana vitrectomy to reduce IOP and perform direct central retinal artery massage [5,6]. Unfortunately, there is no treatment with proven efficacy to date.

Aside from occlusion length, visual outcomes differ depending on the type of CRAO, with arteritic CRAO being the one with the worse prognosis. This is explained because almost all these patients, in addition to CRAO, have associated arteritic anterior ischemic optic neuropathy, resulting in ischemia of both the retina and ONH. In these patients, the early start of high-dose corticosteroid therapy is critical to avoid bilateral blindness [4].

In the case presented, the exact cause of CRAO could not be elucidated. Although the marked decrease in ESR and CRP levels after the onset of high-dose corticosteroid therapy makes arteritic CRAO a plausible diagnosis, elevation of acute-phase reactants might have been related to recent kidney transplantation surgery, and no signs of arteritis were detected in echo-Doppler of temporal arteries. Regarding a non-arteritic etiology, internal carotid artery embolism seems unlikely, as echo-Doppler of supra-aortic vessels did not show significant stenosis. However, a cardioembolic origin might be more probable, given that the patient was affected by auricular fibrillation and was not following full treatment doses of anticoagulation therapy since one week before surgery. As arteritic etiology could not be dismissed, the patient was treated with high-dose corticosteroid therapy, and in relation to a possible embolic cause, full anticoagulation therapy was restored. Sadly, none of these was effective enough and bilateral CRAO resulted in irreversible bilateral amaurosis.

There are a few reported cases of bilateral CRAO, and the majority of them occurred associated with a systemic predisposing condition [7,8]. There is one reported case of bilateral CRAO following lung transplantation surgery, which happened in the context of hemodynamic changes, hypoperfusion, and increased embolic risk [9]. Although non-arteritic CRAO is more common than arteritic CRAO, most bilateral CRAO in elderly patients is believed to be related to temporal arteritis [10].

This case of subsequent bilateral permanent visual loss highlights the terrible consequences of CRAO and how the precise causal mechanism is sometimes hard to establish.

## Conclusions

Embolic CRAO is a rare but devastating complication of non-ophthalmological surgery that must be considered in postoperative patients with visual complaints. Arteritic CRAO should always be considered in patients aged over 50 years, and corticosteroid therapy must be initiated trying to prevent bilateral blindness. According to its significant systemic implications, including increased stroke risk, and given the importance of an early diagnosis, all physicians should be aware of how patients with suspected CRAO must be rapidly referred for both ophthalmological and general evaluation.
